# Circulating enolase 1 as a diagnostic biomarker for early-stage breast cancer

**DOI:** 10.1038/s41698-025-01109-y

**Published:** 2025-10-17

**Authors:** Nikki Salmond, Renata Moravcova, Wing Sum Tam, Karan Khanna, Jason C. Rogalski, Kalan Lynn, Muriel Brackstone, Peter H. Watson, Karla C. Williams

**Affiliations:** 1 Faculty of Pharmaceutical Sciences, University of British Columbia, Vancouver, BC Canada; 2 Proteomics & Metabolomics Core Facility, Life Sciences Institute, University of British Columbia, Vancouver, BC Canada; 3 Lawson Research Institute, London, ON Canada; 4 Department of Surgery, Western University, London, ON Canada; 5 Deeley Research Centre, BC Cancer Agency, University of British Columbia, Victoria, BC Canada

**Keywords:** Biomarkers, Diagnostic markers, Cancer, Breast cancer

## Abstract

Diagnosis of stage 1 breast cancer is challenging as small tumors are often left undetected by conventional imaging techniques. In addition, ~80% of detected breast masses are classified as benign, which means that a large proportion of diagnostic needle biopsies lead to unnecessary psychological stress and medical costs. We investigated circulating extracellular vesicles (EVs) as potential carriers of unique cancer-associated proteins capable of reporting on a breast cancer diagnosis. We isolated EVs from healthy (19), benign (19), and stage 1 breast cancer patient (86) plasma samples using size exclusion chromatography. Mass spectrometry identified 94 significantly changed proteins in the plasma EVs from breast cancer patients. Analysis of a subset of these proteins using a cohort of pre- and post-operative breast cancer patient plasma EVs identified enolase 1 as a promising biomarker. We further validated enolase 1 in a larger patient cohort by high-throughput ELISA of plasma. Enolase 1 was found to be significantly elevated in plasma from stage 1 breast cancer patients compared to healthy and benign individuals, and decreased in post-operative plasma upon tumor removal. Our findings suggest that an enolase 1 liquid blood biopsy could be used to support the detection of breast cancer at the earliest, most treatable, stage.

## Introduction

Early diagnosis of breast cancer is essential for successful treatment outcomes. When breast cancer is diagnosed at an early-stage (stage 1), where the tumor is small and still confined to the breast, the 5-year survival is almost 99.4%. However, in later stage breast cancer where the tumor is larger and/or tumor cells have spread to lymph nodes (stage 3) or distant organs (stage 4), 5-year survival drops to 87.6% and 30.1%, respectively (2014–2020 SEER data, 2024).

Accurate diagnosis of breast cancer at stage 1 can be challenging - the tumor may be too small to palpate or to detect in a routine diagnostic mammogram, while high breast tissue density can increase the chances of false positive mammogram results. A large proportion of masses identified by mammography are benign; this results in many invasive and unnecessary diagnostic tests being carried out (ultrasound and fine needle biopsy) to ascertain whether the mass is benign or cancerous. What is needed is a simple test that could: 1) routinely diagnose breast cancer at its earliest stage and/or 2) be used alongside mammography to distinguish between benign and cancerous lesions. By detecting the majority of breast cancer cases at stage 1, this would shift the breast cancer landscape to the most treatable stage. Distinguishing between breast cancer and benign disease could support a decrease in the use of unnecessary medical procedures and stress to the patient. A simple blood test – a liquid biopsy – represents an ideal platform for the detection of small (<1 cm) breast tumors.

Recent efforts in blood-based biomarker discovery have been focused on identifying circulating tumor cells or tumor-derived DNA in the patient’s blood^1^. While useful, these approaches have significant limitations associated with low abundance necessitating large volumes of blood. Additionally, the procedures can be expensive and technically difficult to complete in a high throughput manner^1^. A circulating protein biomarker is preferable as smaller volumes of blood are required, and assays used in the clinic to quantify proteins are common, rapid, and, thus, likely easier to implement. However, serum or plasma produced from whole blood represents a challenge due to the complexity of the biological fluid, which contains thousands of different proteins. What is more, some proteins are present in high abundance, for example albumin (which represents 50% of all plasma proteins), lipoproteins and immunoglobulins amongst others^2^. Because of this large dynamic range, proteins present in high abundance can make the identification of new diagnostic tumor biomarkers that are likely present in low abundance challenging^3^. Protein biomarker discovery in plasma requires the immunodepletion of 7–14 of the most abundant proteins found in plasma. But low abundant biological biomarkers are still challenging to identify as the immunodepletion process can also remove proteins of interest^2,4^. The use of multi-nanoparticle enrichment of plasma-protein coronas for mass spectrometry (SEER), has shown success in improving the depth and breadth of protein identification from plasma while avoiding immunodepletion strategies, however there are significant costs associated with the process^5^.

An alternative strategy for blood-based protein biomarker discovery is to enrich and analyze tumor-derived extracellular vesicles (EVs)^6–8^. Tumor cells constantly shed fragments of themselves (EVs) into the extracellular environment and circulation^9^. EVs are lipid bilayer-limited biological nanoparticles (50 nm–1 µm) released by all cell types to support cell-cell communication in healthy homeostasis and pathological diseases, such as cancer. Cells package protein, DNA, RNA, lipids, and metabolites into EVs meaning that EV contents can reflect the parental cell molecular phenotype^9^. Cancer cells release more EVs than corresponding healthy cells and have a unique protein content signature^10–13^. This is why enrichment of plasma derived EVs, a proportion of which will be breast tumor derived, represents a unique opportunity to increase the chances of detection of novel breast cancer biomarkers. If we can create a protein fingerprint of breast cancer patient EVs, we may be able to identify novel diagnostic biomarkers that can be used directly from plasma to diagnose breast cancer at its earliest stage.

Here we describe the development of a method for the isolation and mass spectrometry analysis of EV-associated proteins which was applied to a unique discovery cohort of healthy (*n* = 19), benign (*n* = 19), and stage 1 breast cancer (*n* = 77) patient plasma samples. In addition, we assembled a small cohort of matched plasma samples from stage 1 breast cancer patients both pre-operative (tumor in-place *n* = 9) and post-operative (tumor removed, *n* = 9). Our work identified several proteins elevated in early-stage breast cancer as compared to individuals that are healthy or have a benign breast mass. Using the pre-operative and post-operative plasma samples to determine which biomarkers decreased after tumor removal, enolase 1 was selected for further validation. Enolase 1 was then validated as a diagnostic biomarker for breast cancer of all stages including stage 1, where five-year survival is almost at 100%, using a high throughput and ELISA assay that would be amenable to a clinical application.

## Results

### Size exclusion chromatography is the optimal method for EV isolation from plasma

Different methods for EV isolation were compared to identify the optimum technique for EV isolation and downstream mass spectrometry proteomic analysis for biomarker discovery. Size exclusion chromatography (SEC), precipitation, and the ExoQuick**®-**Ultra kit were all used to isolate EVs from 500 µl plasma. Western blot showed that SEC isolated CD63 and CD9 positive EVs, and precipitation using PEG before SEC did not improve EV yield (Supplementary Fig. 1A, D, full western blots can be found in Supplementary Fig. 2). Precipitation using PEG and ExoQuick**®** did not isolate significant amounts of EVs but did enrich for contaminants such as albumin. Any protocol involving ExoQuick**®-**ultra, depleted the lipoprotein ApoA1 content of EV preparations. However, ExoQuick**®-**Ultra followed by SEC decreased the protein concentration to a point that any downstream processes would be difficult (Supplementary Fig. 1B). Electron microscopy indicated that ExoQuick**®**-Ultra samples contained more protein contamination than SEC EV samples (Supplementary Fig. 1C).

The protein profile of 20 µg of EVs isolated by SEC, ExoQuick**®**-Ultra and ExoQuick**®**-Ultra-SEC was further interrogated by mass spectrometry. Gene Ontology (GO) analysis (WebGeSalt, 2024) of the identified proteins showed that SEC, ExoQuick**®-**Ultra, and ExoQuick**®-**Ultra-SEC EVs were all enriched in proteins that localized to cellular compartments involved in EV biogenesis pathways (Supplementary Fig. 3A). Furthermore, each technique yielded EV preparations with unique protein compositions as displayed by the Venn Diagram (Supplementary Fig. 3B). From the top 100 EV proteins identified in published mass spectrometry experiments (Vesiclepedia 2024), 42 were identified in EV preparations isolated by SEC, compared to only 26 by ExoQuick**®-**Ultra and ExoQuick**®-**Ultra-SEC EV preparations (Supplementary Table 1). Additional EV proteins, not included in the top 100 list, were identified exclusively in SEC preparations including luminal proteins ALIX and syntenin, several Rab proteins including Rab27b, flotillin, and several integrins including integrin β1 (Supplementary Table 1). To further validate our SEC-isolated EV preparations, western blot analysis of CD9 was performed on EVs isolated from the plasma of eight healthy individuals; CD9 was found to be reproducibly present in all eight patient EV samples (Supplementary Fig. 1D).

Overall, the results indicate that SEC efficiently isolates EVs with a sufficient protein concentration for downstream applications, and was the technique that most efficiently enriched for EV-proteins relative to the other methods tested. It was concluded that SEC would be the most appropriate method for the isolation of EVs from clinical plasma for downstream mass spectrometry analysis.

### Mass spectrometry analysis of stage 1 breast cancer patient EV-associated proteins

**Fig. 1:**
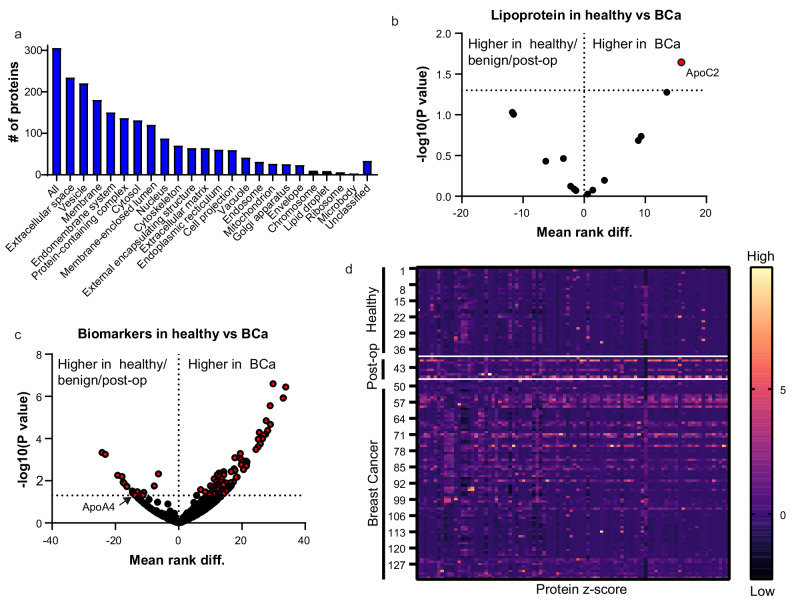
Mass spectrometry analysis of healthy, benign, and stage 1 breast cancer patient plasma-derived EVs. **a** All proteins identified in the mass spectrometry data were run through GO analysis software (WebGeSalt, 2024) and the cellular compartments from which the identified proteins arose were analyzed. **b** Apolipoprotein mass spectrometry data was transformed to give any zero values a value of 0.000001, and a multiple Mann–Whitney T-test was used to identify any apolipoproteins that were statistically significantly different in the healthy (*n* = 19)/benign (*n* = 19)/post-operative (*n* = 9) group compared to breast cancer (*n* = 77)/pre-operative (*n* = 9) cohort. **c** Mass spectrometry data was transformed and analyzed using GraphPad Prism and a volcano plot generated to identify any statistically significant proteins elevated in the breast cancer (*n* = 77)/pre-operative (*n* = 9) patient plasma samples as compared to healthy (*n* = 19)/benign (*n* = 19)/post-operative (*n* = 9) plasma samples. A multiple Mann–Whitney T-test was done to ascertain *p*-values. **d** Heat map generated in GraphPad prism gives a visual representation of the z-score (=observed score − mean of the sample/standard deviation) of identified proteins in healthy (*n* = 19)/benign (*n* = 19) individuals versus post-operative (*n* = 9) and breast cancer (*n* = 77 + *n* = 9 pre-operative) samples. Yellow/orange colors indicate a protein that is more abundant, with decreasing abundance represented through pink, purple and black.

EVs were isolated by SEC from up to 500 µl (0.8 µm filtered) of plasma from19 healthy, 19 benign, 9 post-operative, and 86 stage 1 breast cancer patient (including 9 matched pre-operative). Supplementary Fig. 4 shows that the 0.8 µm filtration removed platelets from plasma samples while retaining CD9 positive EVs, rendering our plasma samples platelet depleted. This eradicated the unwanted possibility of platelet activation and release of excess platelet-derived EVs during EV isolation, or upon thawing of plasma. Clinical cohort information is shown in Supplementary Table 2 and a report on cohort blood collection and processing is provided (MIBlood-EV supplemental reporting document)^14^. After protein quantification, 20 µg of EVs were lysed in 2x volume RIPA buffer and proteins were isolated by acetone precipitation. Proteins were reduced and digested into peptides using trypsin and 500 ng was used for LC/MS mass spectrometry. The resultant 316 protein identifications were analyzed by GO analysis (WebGeSalt 2024) which showed that the protein dataset was enriched in proteins that derive from cellular compartments involved in EV biogenesis (Fig. 1a).

To ensure standardized quantitation between sample preparations and mass spectrometry runs, each sample was spiked with 200 fmol of a yeast glutathione reductase (GSHR) protein. The quantitative intensity value (iBAQ) was normalized relative to GSHR for each identified protein. Potential protein biomarkers of early-stage breast cancer were identified as those that were significantly elevated in stage 1 breast cancer patients as compared to healthy and benign controls.

Our mass spectrometry data identified apolipoproteins as some of the most abundant proteins. This was expected as apolipoproteins are abundant in plasma and, due to their size and density, co-isolate with EVs by SEC. As there is no strong link between circulating lipid components and risk of developing breast cancer, as demonstrated by for ApoA1 and ApoB^15,16^, these common contaminants of EV preparations would not be expected to change significantly between healthy, benign and breast cancer patient plasma samples. Analysis of apolipoprotein levels, as determined by mass spectrometry, found similar abundances in breast cancer patient plasma samples as compared to healthy, benign and post-operative individual plasma samples, with the exception of ApoC2 and ApoA4. ApoC2 was found to be significantly higher in breast cancer compared to healthy/benign/post-operative (Fig. 1b) samples, and ApoA4 was found to be significantly lower in breast cancer compared to healthy/benign/post-operative samples (Fig. 1c).

Next, we evaluated the protein expression profiles of plasma-isolated EVs from healthy, benign, and breast cancer patients. Seventy three proteins were found to be significantly elevated and 19 were significantly reduced in breast cancer patient plasma derived EVs as compared to healthy/benign EV samples (Fig. 1c and Supplementary Table 3). Supplementary File 1 details all identified proteins and their average iBaq values in each patient cohort. The potential significant biomarkers are listed in Supplementary Table 3 with their *p*-values. GO analysis showed that proteins significantly elevated in breast cancer patient plasma samples have a strong association with vesicles, membranes and the extracellular space. KEGG pathway analysis indicated that proteins involved in the coagulation and the complement system are elevated in breast cancer patient plasma, as are proteins associated with pathways that are supportive of cell migration, amino acid biosynthesis and glycolysis (Supplementary Fig. 5). The protein absence or presence in each plasma sample (low to high) is represented in the form of a heat map (Fig. 1d). The heat map shows that the protein profile of healthy (*n* = 19)/benign (*n* = 19) EVs is different as compared to stage 1 breast cancer EVs (*n* = 86).

**Fig. 2:**
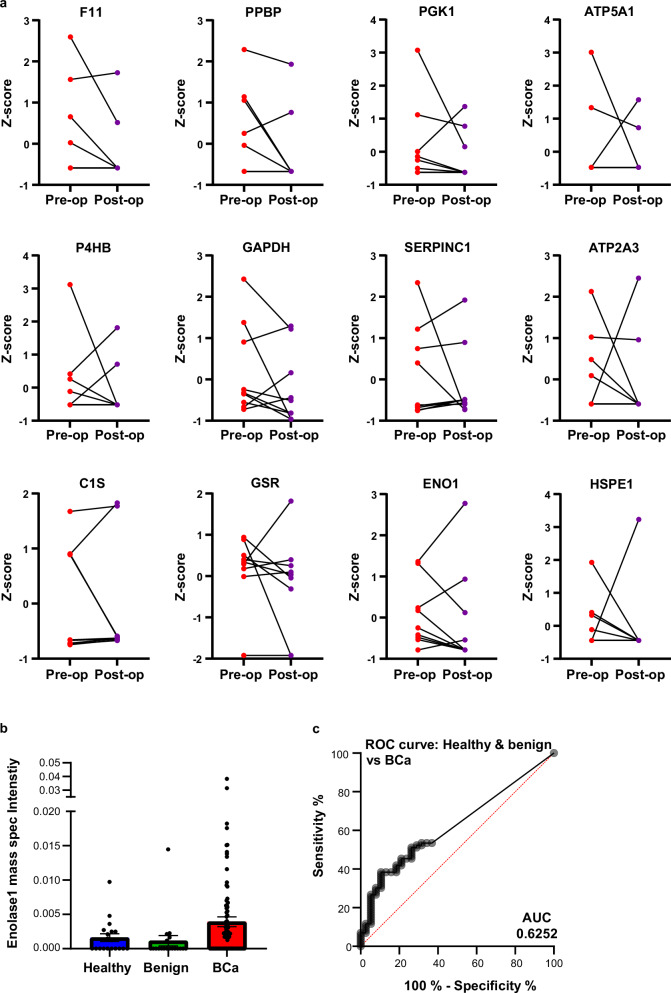
Mass spectrometry identification of EV-associated enolase 1 as a biomarker for the diagnosis of stage 1 breast cancer. **a** Proteins identified as being significantly elevated in breast cancer patient plasma-derived EVs by mass spectrometry were analyzed in patient plasma samples taken pre- and post-operative (*n* = 9). The twelve proteins that had the highest fold change in average circulating levels post-operative were selected as potential biomarker candidates. **b** Detection of enolase 1 in patient-derived EV plasma samples by mass spectrometry was analyzed, iBaq values were plotted from healthy, benign and breast cancer patient samples. Kruskal–Wallis test was performed to determine significance. **c** The plotted iBaq values were used to construct a receiver operating characteristic (ROC) curve comparing healthy/benign enolase 1 levels to breast cancer patient enolase 1 levels to ascertain the specificity and sensitivity of enolase 1 as a diagnostic biomarker. Graphs constructed in prism. Sample numbers: Healthy (19), Benign (19), breast cancer (*n* = 86).

To refine the list of significant proteins elevated in breast cancer patient plasma-derived EVs, a unique patient cohort of plasma taken from patients before tumor removal (pre-operative), and then again at least 1 month after tumor removal (post-operative) was utilized. Any biomarkers associated with the presence of a tumor were expected to decrease in the post-operative patient plasma samples. Biomarkers of interest that were significantly higher in stage 1 breast cancer compared to healthy/benign were analyzed in the sub-set of pre-/post-operative samples (Fig. 2a). For this, z-score normalization was applied to the mass spectrometry data which represents the number of standard deviations that the pre/post-operative value deviates from the mean of the group. While some biomarkers decreased post-surgery, others remained the same or even increased suggesting that those biomarkers had low specificity in breast cancer detection and were therefore excluded from further analysis. The 12 biomarkers that displayed the largest decrease in post-operative patients, post-tumor removal, are shown in Fig. 2a from nine patient matched pre-operative and post-operative plasma samples. In our previous work, we identified that platelet-related proteins may not make useful biomarkers due to person-to-person and plasma collection variability^17^. On this basis we excluded F11, PPBP, and SerpinC1 from further investigation. We also removed C1S which is a protein involved in the complement system. While potentially an interesting biomarker, interpretation of complement proteins as biomarkers can be difficult in terms of patient to patient variability and exposure to pathogens. After a literature search of the remaining proteins, enolase 1 was chosen for further investigation due to the rich literature suggesting that it has a biological role in breast cancer cell invasion, is associated with EVs, and plays a role in tumorigenic phenotype propagation^18–20^.

### Validation of enolase 1 as a diagnostic biomarker for stage 1 breast cancer

The mass spectrometry data was further analyzed and enolase 1 was shown to be elevated in plasma-derived EVs from breast cancer patients as compared to healthy or benign individuals (Fig. 2b). The receiver operating characteristic (ROC) area under the curve (AUC) was 0.6252 and indicated that, while low, there was potential for discrimination between healthy/benign and breast cancer. The diagnostic test specificity (ability to diagnose a patient with breast cancer) of 81.58% gives a sensitivity (correctly identifying patients that do not have breast cancer) of 41.86% (Fig. 2c). This means the likelihood of a false positive result is higher than would be acceptable for a diagnostic test. While mass spectrometry is a powerful tool for biomarker discovery, the quantitative analysis of low abundant proteins in patient plasma derived-EVs by mass spectrometry is not an ideal clinical test due to the variability inherent in multi-step sample preparation, workload and equipment needs. Therefore, we aimed to evaluate enolase 1 levels directly in-patient plasma using a high throughput and sensitive technique suitable for the clinic.

**Fig. 3:**
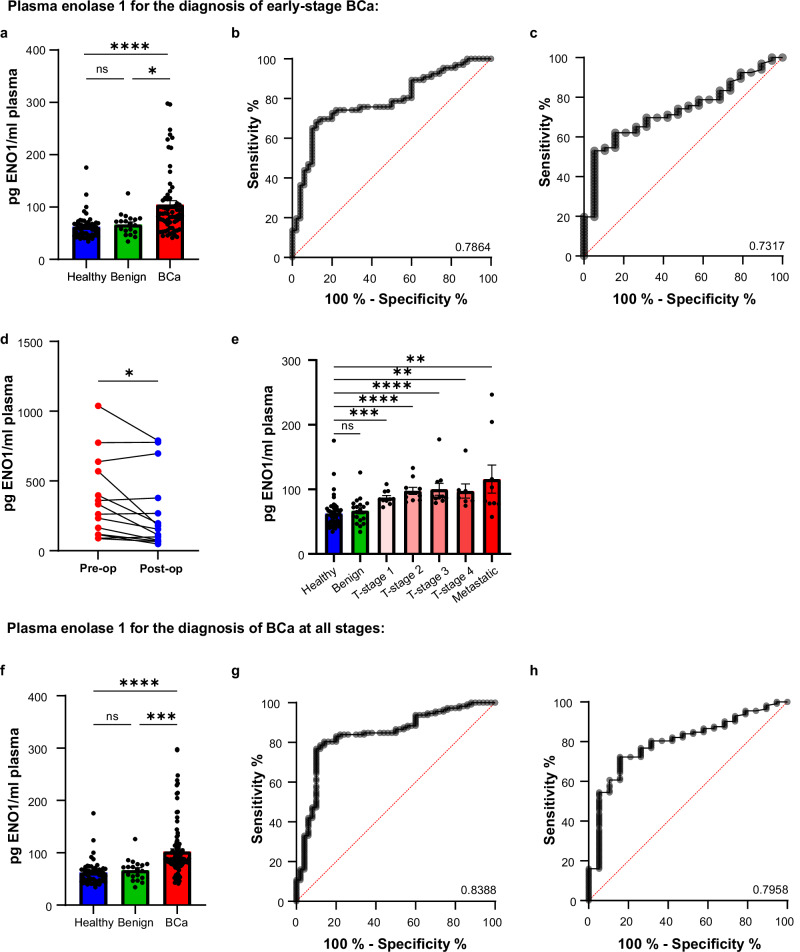
Enolase 1 is an effective biomarker for stage 1 breast cancer and can be analyzed directly from patient plasma samples using high throughput ELISA*.* **a** ELISA of 25 µl patient plasma for enolase 1. Healthy (*n* = 50), benign (*n* = 19), breast cancer (*n* = 66). ROC curves show the sensitivity and specificity of enolase 1 at identifying stage 1 breast cancer in healthy vs breast cancer patient plasma (**b**) or benign vs breast cancer patient plasma (**c**). **d** Matched patient plasma samples taken from breast cancer patients before they had an operation to remove their breast tumor, and again at least 25 days after. Plasma samples were used in ELISA to quantify the amount of enolase 1. (*n* = 14). **e** Breast cancer patient plasma of different tumor stage (size) from 1 to 4 and patients with metastatic disease were analyzed for enolase 1 by ELISA. T1 (*n* = 10), T2 (*n* = 10), T3 (*n* = 10), T4 (*n* = 7), metastatic (*n* = 9). **f** All stage 1 and later tumor stage 2–4 and metastatic breast cancer patient plasma samples were pooled to ascertain the diagnostic potential of enolase 1 at any breast cancer tumor stage. ROC curves were constructed to determine enolase 1 specificity and sensitivity for the identification of patients with breast cancer in healthy (*n* = 50) vs breast cancer (*n* = 112) (**g**) and benign (*n* = 19) vs breast cancer (*n* = 112) (**h**) cohorts.

Using an Enzyme Linked Immunosorbent Assay (ELISA), enolase 1 levels in 50 healthy, 19 benign, 113 breast cancer, and 14 matched pre-operative and post-operative samples were analyzed. Samples that had remaining plasma from EV isolation for mass spectrometry were used (66 samples stage 1) alongside new matched pre-operative and post-operative samples and samples of varied tumor stage. ELISA confirmed that enolase 1 was significantly elevated in breast cancer patient plasma samples as compared to healthy or benign individuals (Fig. 3a). A ROC curve showed that ELISA analysis of plasma improved upon the mass spectrometry analysis of plasma-derived EVs for correctly identifying patients with breast cancer (AUC healthy:breast cancer 0.7864, AUC benign:breast cancer 0.7317). Setting the test sensitivity to 80%, the specificity was 72.73% (healthy:breast cancer) and 62.12% (benign:breast cancer). The ELISA platform demonstrated fewer false positives and, thus, improved sensitivity as compared to mass spectrometry analysis of patient derived EVs (Fig. 3b, c).

A new set of the unique pre-operative and post-operative plasma samples (*n* = 14) was used for further validation of enolase 1 as a tumor associated biomarker (Supplementary Table 4). ELISA analysis confirmed that enolase 1 levels in patient-plasma decreased after tumor removal (Fig. 3d). However, plasma enolase 1 was not able to differentiate between patients with breast cancer of different tumor sizes (1 (<2 cm), 2 (>2 cm <5 cm), 3 (>5 cm), 4 (any size tumor growing into chest wall or skin)) or with metastatic disease (Fig. 3e). Nevertheless, the ROC curve indicates excellent specificity and sensitivity of enolase 1 for the diagnosis of breast cancer at all stages (Fig. 3f). When compared to healthy individuals, if the test had a sensitivity of 80.36%, the specificity was 86% (healthy:breast cancer) and 68.42% (benign:breast cancer) (Fig. 3g, h). The enolase 1 levels in plasma samples were also analyzed in the context of different breast cancer subtypes (ER, PR, HER2, triple negative), clinical grade, tumor grade and recurrence. There was no statistically significant difference in plasma enolase 1 in patients with different breast cancer stages or subtypes (Supplementary Fig. 6).

## Discussion

Proteomic analysis of EVs from the plasma of healthy, benign, and breast cancer patients revealed enolase 1 to be a potential biomarker for the diagnosis of stage 1 breast cancer. EV isolation is a time-consuming and multi-step process, making the use of EVs in a clinical test difficult. Therefore, we analyzed enolase 1 directly from platelet-depleted plasma using a high-throughput ELISA assay that could be easily translated into a clinical setting. The clinical ELISA data indicated that enolase 1 could be a useful diagnostic biomarker for not only breast cancer of all stages, but most importantly, early-stage breast cancer where the chances of curative treatment are highest. Given that enolase 1 can be secreted, absorbed onto plasma membranes, and has been identified as an EV component^18,21,22^, our analysis of enolase 1 in plasma would encompass all these forms. Cell surface associated enolase 1 could result in enolase 1 positive ectosomes, and/or secreted enolase 1 could absorb onto EV surfaces as a component of the EV corona. We have previously demonstrated that EV corona-associated proteins can have potential diagnostic utility in cancer^17^. Our current study further supports this, suggesting that bona fide EV constituents as well as EV-associated proteins have utility as biomarkers in health and disease.

Of the three enolase isoforms, enolase 1 is ubiquitously expressed in most tissues and the function is dependent upon post-translational modifications which determine the localization of enolase 1 in the cell^23^. In the cytoplasm, enolase 1 is a phosphopyruvate hydratase and is a glycolytic enzyme in the glycolysis pathway^24^. In the nucleus, a truncated version of enolase 1 binds and suppresses the myc promotor resulting in tumor-suppressive properties^25^. Additionally, enolase 1 is documented to be secreted into the extracellular space either as a soluble protein or associated with EVs, where it can bind to other cell types and act as a plasminogen binding receptor supporting pro-invasive matrix modification for immune cell, pathogen, and cancer cell migration^18,26–28^. In breast cancer, enolase 1 has been associated with cytoskeletal re-organization, migration, invasion and proliferation^18–20^, as well as being indicative of successful treatment responses^29^. Enolase 1 has also been reported to be associated with breast cancer-derived EVs which support pro-tumorigenic migration and invasion^18,30^. The biological function and EV localization of enolase 1 reported in the literature aligns with our findings of elevated levels in breast cancer patient plasma.

In breast cancer clinical samples, enolase 1 expression in stage 1 and 2 breast tumors has been documented to be a good prognostic indicator and associated with the presence of tumor infiltrating lymphocytes^31^. Furthermore, when S100A9 was overexpressed in HER2-positive tumor tissues, enolase 1 expression was also upregulated^32^. Previously, enolase 1 has been investigated as a potential liquid biopsy biomarker. For example, enolase 1 was reported to be elevated in breast cancer patient saliva^33^. Another study reports decreased detection of enolase 1 in breast cancer plasma compared to healthy individuals, but the patient cohort was small (10 healthy and 10 breast cancer)^34^. Our study used a much larger cohort giving more power and statistical significance to the results. Supporting our results, a different study found elevated enolase 1 in the plasma of canines with mammary carcinoma, although also with a smaller sample size^35^. Additionally, enolase 1 has been detected in the lymphatic fluid of tumor-draining lymph vessels^36^. Most published studies support our data in that they report an elevated secretory profile of enolase 1 in breast cancer patients. Our study is the largest so far to confirm that enolase 1 is elevated in breast cancer patient plasma compared to healthy individuals, and furthermore, the first to report that plasma enolase 1 can distinguish between benign and cancerous growths.

EVs constitute a small portion of all particles in the blood (0.0002%), and rare tumor-derived EVs are represented by only 0.0000003%/1 cm^2^ tumor^37^. The ‘compartmental model’ of tumor EV release rates into blood suggests that tumors around 1 mm^3^ could feasibly be detected via EVs present in 1 ml of blood. However, analysis of EVs by methods such as ELISA are thought to not be sensitive enough to detect tumors <1 cm^3^, posing some limitations for biomarker discovery, especially at early stages where the tumor is very small^37,38^. The success of our biomarker discovery study, regardless of the rarity of circulating EVs, may be attributed to careful EV enrichment and purification which separated EVs away from the very abundant proteins (immunoglobulins, albumin) in whole plasma. This would decrease the dynamic range between the most abundant proteins present in plasma as compared to the ‘rarer’ tumor biomarkers, increasing the probability of detection of rare tumor-derived events in the EV preparation as compared to whole plasma mass spectrometry. Additionally, we previously reported that the secreted breast tissue biomarker mammaglobin-A transiently associated with platelet derived EV membrane coronas in plasma^17^. We hypothesise that enolase-1 in the blood is present in both soluble and EV corona-associated forms. Thus, the EV protein corona may be an interesting proteome to exploit to gain a better understanding of the tumor secretome and identify new diagnostic biomarkers.

There are some considerations to address for the analysis of the next larger patient cohort. The current cohort of plasma samples were acquired through two different biobanks and prepared using both EDTA and Heparin either at room temperature or 4 °C. While centrifugation of plasma at 4 °C is reported to activate platelets^39^, the effect of heparin on platelet activation and platelet derived EV release is concentration dependent and ambiguous^40–42^. The ELISA data indicates that the detection of enolase 1 was not significantly affected by whether heparin or EDTA anticoagulant was used (Supplementary Fig. 7). However, if we were to validate these results in a larger cohort, it would be imperative to ensure all plasma was collected using the same protocol, temperature, and anticoagulant.

An AUC of 0.8 is generally considered to indicate good diagnostic test accuracy that may be clinically useful, depending on the application. In our plasma-enolase 1 ELISA for stage 1 breast cancer, the AUC was found to be 0.7864 when comparing healthy to breast cancer patients. However, when comparing benign to breast cancer patients, the AUC dropped to 0.7317. If sensitivity was set to correctly identify 80% of patients with breast cancer, we are only correctly identifying 72.73% (healthy) and 62.12% (benign) of patients that do not have breast cancer. Furthermore, the lack of specificity of enolase 1 to breast cancer could also be an issue as enolase 1 is ubiquitously expressed and a potential biomarker for other cancers^43–45^. Both of these factors indicate that a test using enolase 1 alone for the diagnosis of stage 1 breast cancer, would 1) have unacceptable numbers of false positives, leading to unnecessary biopsies being carried out, and 2) not have the specificity to diagnose only breast cancer. To rectify this, we anticipate that using other biomarkers alongside enolase-1 would be important to further improve the sensitivity and specificity of the test. Further investigation of other proteins identified by mass spectrometry would be an important next step.

Alternatively, an enolase 1 test could be used in combination with mammography to distinguish between patients with benign growths and cancerous growths. As up to 80% of breast growths are benign, many unnecessary needle biopsies are carried out every year. A blood test that would accompany imaging could help direct the physician’s decision as to whether the biopsy is necessary and decrease the number of unnecessary biopsies carried out. Finally, as the test was capable of detecting decreases in circulating enolase 1 after tumor removal, using enolase 1 to monitor for recurrence may prove to have benefit. This may be a worthwhile consideration in patients at high risk of recurrence, such as TNBC patients.

## Methods

### Plasma for EV isolation technique optimization and comparison

Pooled human female plasma (age 40–60) was collected in K2EDTA tubes (Innovative Research). Blood was pooled and processed to isolate plasma using apheresis.

### Clinical plasma cohort

Plasma samples from healthy, benign and breast cancer patients were obtained by the BC Cancer Biobank (Victoria BC) and London Tumor Biobank (London Ontario). Blood was collected in K2EDTA tubes (10 ml) or Na/Heparin tubes (4 or 6 ml) and plasma was isolated by centrifugation at 2500 × *g*. Plasma was centrifuged for 15 min at 4 °C (London Tumor Biobank) or 2500 × *g* for 10 min at room temperature (BC Cancer Biobank). Healthy (*n* = 50) and benign (*n* = 19) samples were obtained with patient consent in accordance with the London Health Sciences guidelines and ethical considerations (REB# 116689). Samples were taken from breast cancer patients ranging from tumor stage (1–4) with a focus on stage 1 (*n* = 86), stage 2 (*n* = 10), stage 3(*n* = 10) and stage 4 (*n* = 7) or with metastatic disease (*n* = 9). Matched plasma samples obtained pre-operative (*n* = 23) and post-operative, after tumor removal, (*n* = 23) were also used in the study. Samples were obtained, stored in liquid nitrogen, shipped on dry ice, and stored at −80 °C upon arrival. This study is compliant with all relevant ethical regulations on the use of human plasma and all research was performed in accordance with the declaration of Helsinki. Study approval was obtained by the institutional review board of UBC (IRB#H17-01442).

### Extracellular vesicle isolation: plasma preparation

Plasma samples were thawed quickly in cold water and filtered using a 0.8 µm filter (SLAA0335B Sigma and 431221 Corning). For EV isolation and mass spectrometry method optimization, 500 µl of pooled plasma (Innovative Research) was used. For the isolation of EVs from the clinical sample cohort plasmas, 500 µl of plasma used for the majority, if a smaller sample volume was used a total volume of 500 µl was ensured using PBS.

### Extracellular vesicle isolation: size exclusion chromatography

SEC columns (qEV original 70 nm, 2021 version – Izon) were equilibrated to room temperature and washed with 20 ml PBS prior to experiment. For EV isolation, 500 µl plasma was applied to the SEC column and the flow though collected immediately. As the plasma passed through the column, PBS was added to maintain continuous flow. Three milliliters of flow through was collected and discarded followed by 2.5 ml of EV fractions. EVs were concentrated at 3000 × *g* in 10-min intervals using a 10 kDa MWCO Amicon centrifugal concentrator (Millipore) to ~100 µl.

### Extracellular vesicle isolation: precipitation with PEG-6000

500 µl plasma was incubated with 500 µl PEG-6000 (20% w/v PEG-6000 Sigma Aldrich, 200 mM NaCl, 100 mM EDTA, 200 mM Tris-HCL, pH 7) for 1 h 4 °C. The precipitate was then pelleted by a 3000 × *g* spin for 10 min. The precipitate pellet was re-suspended in 500 µl PBS. If necessary, precipitation was followed by SEC described above.

### Extracellular vesicle isolation: precipitation with ExoQuick®

EVs were precipitated by incubation of 500 µl plasma with 134 µl ExoQuick® (SBI) for 30 minutes at 4 °C. The precipitate was pelleted by centrifugation at 3000 × *g* for 10 min and the precipitate was re-suspended in 500 µl PBS.

### Extracellular vesicle isolation: ExoQuick®-Ultra

Precipitation of EVs using ExoQuick® was done as described above, and the precipitated pellet was re-suspended in 200 µl Buffer B and 100 µl Buffer A and manufacturer’s instructions (SBI) were followed for the EV clean-up. One Ultra column was used per 250 µl plasma precipitate.

### Electron microscopy

EVs were fixed in 2% electron microscope grade paraformaldehyde and adsorbed onto Formvar/carbon coated nickel or copper 200 mesh grids for 2 min (Ted Pella/Electron Microscopy Sciences respectively). Uranyl acetate (1%, pH 4.6, 30 s, Fisher Sci) was used to negatively stain EVs, grids were blotted and air dried. EVs were imaged using a Helios NanoLab 650 in scanning transmission mode (ThermoFisher, Systems for Research, Kanata, ON, Canada) at 30 kV using the bright field imaging mode.

### EV protein quantification

Isolated EV protein content was quantified using a BCA rapid gold protein quantification kit (Thermo Fisher Scientific) according to manufacturer’s instructions.

### Mass spectrometry: sample preparation

Twenty micrograms of proteins from each EV sample were subjected to acetone precipitation^46^. Protein pellets were re-suspended in 6 M urea/2 M thiourea. Samples were reduced in dithiothreitol solution (1 µg/50 µg protein) and incubated for 30 min at room temperature, followed by alkylation with iodoacetamide (5 µg/50 µg protein) and incubation for 20 min at RT in the dark. Proteins were digested with 0.25 µg Trypsin/LysC enzyme for 3 hours at RT. Next, samples were diluted by four volumes of 50 mM ammonium bicarbonate (pH ~8), followed by adding 0.2 µg Trypsin (Promega) for 19 h at room temperature^47^. Trypsin activity was quenched by acidification to pH <2.5 resulting in supernatants of peptides. Samples were cleaned up via STAGE-Tip purification^48^, briefly: each of the samples was forced through a conditioned and equilibrated homemade column with 11 mm of C18 packing, washed with 0.1% TFA twice, and eluted into clean tubes by buffer containing 40% ACN, 0.1% TFA, then dried down.

### Mass spectrometry: LC/MS analysis

Prior to LC-MS/MS analysis^49^, each sample was reconstituted in 2% ACN, 0.1% formic acid and measured for final concentration at A205 using NanoDrop One (ThermoFisher) to inject. Sample (500 ng) was mixed with 200 fmol of internal GSHR yeast glutathione reductase standard. The digest was separated using EasyLC 1200 HPLC (ThermoFisher Scientific) with Aurora Series Gen2 (CSI) analytical column (25 cm × 75 μm 1.6 μm FSC C18, with Gen2 nanoZero and CSI fitting; Ion Opticks, Parkville, Victoria, Australia) heated to 50 °C (using tape heater, SRMU020124, Omega.com, and in-house build microprocessor temperature controller), and coupled to Impact II Qtof (Bruker Daltonics) operated in DDA mode. A standard 90 min gradient was run from 5% B to 13% B over 45 min, then to 35% B from 45 to 90 min, then to 90% B over 2 min, and held at 90% B for 13 min. Before each run, the analytical column was conditioned with 4 column volumes of buffer A. Where buffer A consisted of 0.1% aqueous formic acid and 2% acetonitrile in water, and buffer B consisted of 0.1% formic acid in 80% acetonitrile. The EasyLC thermostat temperature was set at 7 °C. The analysis was performed at 0.4 μL/min flow rate. The Quadrupole – Time of Flight Mass Spectrometer (Impact II; Bruker Daltonics, Germany) was set to DDA auto-MS/MS scanning 200–2000 m/z. The capillary voltage was set to 1900 V, nanoBooster with methanol to 0.25 bar, drying gas to 3 L/min, and drying temperature to 150 °C. The MS and MS/MS spectra were acquired from m/z 200–2000 (at 5 Hz rate) with active focus fragmenting of the 20 most abundant ions (one at the time at 18 Hz rate) after each full-range scan. The active exclusion was enabled with a 0.4 min release. The collision energy ranged from 23 eV to 65 eV, depending on ion mass and charge^50^. Isolation widths were set to 2–3 Th. Parent ions were then excluded from MS/MS for the next 0.4 min and reconsidered if their intensity increased more than 4 times. Singly charged ions were excluded. Mass accuracy: error of mass measurement is typically within 5 ppm and is not allowed to exceed 10 ppm. For calibration, the ions of Agilent ESI-Low Tuning Mix ions were selected (m/z [Th]: 622.0290; 922.0098; 1221.9906). Impact II was run with OTOF Control v. 4.1 (Bruker). LC and MS were controlled with HyStar 4.1 (4.1.21.2, Bruker).

### Mass spectrometry: data search

Acquired data were searched using MaxQuant^51^ (v. 2.0.3.0), and LFQ intensities extracted and normalized using MaxLFQ^52^ algorithm^52^. Protein sequence database Homo sapiens from Uniprot (reviewed sequences only; downloaded on January 15, 2021), common contaminants protein, and GSHR_YEAST Glutathione reductase protein, containing in total 20431 sequences were used. For analysis, precursor mass tolerance was set up to 20ppm, and 30ppm for fragment mass. Enzyme specificity was set to “trypsin”, with up to 2 missed cleavages allowed. The final data-table was filtered to 1% false discovery rate. A total of 336 proteins were identified based on peptide analysis; 20 were contaminant proteins and removed from the dataset prior to statistical analysis. Two samples (*n* = 1 healthy and *n* = 1 breast cancer) were removed from the dataset prior to statistical analysis due to low GSHR peptide counts. The mass spectrometry data was deposited to the ProteomeXchange via the MassIVE (Mass Spectrometry Interactive Virtual Environment) partner repository^53^ with the dataset identifier PXD060401.

### Nanoscale flow cytometry

As previously reported^17^, 10 µl plasma was incubated with CD9-CF405M primary antibody (1 µl ab123624) or an isotype control (0.5 µl ab126036) for 30 min, room temperature in the dark. Plasma-antibody mixture was re-suspended in 300 µl PBS and analyzed on the CytoFLEX S flow cytometer using violet side scatter as the trigger (detection 1024) and BV (gain 200) on a slow flow rate for 40 s.

### ELISA

For quantification of enolase 1 in 25 µl of 0.8 µm filtered plasma samples we used the human enolase 1 ELISA kit (abcam ab181417) according to manufacturer’s instructions. Samples were tested in duplicate or singlet depending on how much sample and space on the ELISA plate was available.

### Western Blot

EV preparation (experiment dependent amount), was mixed with 4x loading buffer and 10x reducing agent if appropriate. CD63 and CD9 were blotted in non-reducing conditions. After boiling at 95 °C for 10 min, samples were loaded onto 10 or 15 well NuPAGE Bis-Tris gels (Thermo Fisher Scientific) and proteins separated by molecular weight using MES running buffer (1 M MES, 2% SDS, 1 M Tris Base, 20 mM EDTA) at 220 V for 30 min. Proteins were transferred onto 0.45 µm nitrocellulose membrane (BioRad) at 30 V for 90 min using transfer buffer containing 10% methanol (190 mM glycine, 25 mM Tris Base). Membranes were blocked in 5% milk for 30 min, and proteins were probed using the appropriate primary antibody in 1% milk overnight. After three 10-min washes with agitation in TBS-T (20 mM Tris base, 160 mM NaCl, 0.1% Tween), membranes were incubated with a secondary antibody IRdye® 680RD or 800CW (LI-COR 1:10,000) in 1% milk for 30 min protected from light. Membranes were washed again 3 times for 10 min with agitation in TBS-T in the dark. Membranes were then imaged using LI-COR Odyssey®. Images were handled using LICOR Image Studio Lite. All steps were done at room temperature other than the overnight primary antibody incubation which was at 4 °C. Antibodies: CD63 (Abcam: ab59479 1:1000), ApoA1 (Thermo MA1-83002 1:2500), CD9 (Millipore: CBL162), Albumin (Abcam: ab10241 1:2500).

### Statistical analysis

All data was handled using Microsoft Excel and analyzed using GraphPad Prism (10.3.2). The distribution of the data was tested for normality and the appropriate corresponding parametric or non-parametric T-test or One-way ANOVA test was used to determine data significance. For the proteomics data, all data was normalized relative to the run-specific intensities to the spiked in standard protein Yeast GSHR (P41921) and iBAQ^54^ values used for further analysis. Data was transformed to ensure all zero values were assigned a value of 0.000001. Subsequently a multiple Mann–Whitney test was used to determine the mean rank difference and the significance of each identified protein in healthy/benign/post-operative samples versus breast cancer samples (or different combinations therewith). The mean rank difference and p-values were used to generate a Volcano plot *(*p* ≤ 0.05). **(*p* ≤0.01), ***(*p* ≤ 0.001), ****(*p* ≤ 0.0001).

## Supplementary information

Supplementary Information

Study Information

Supplementary Data

## Data Availability

All data needed to evaluate the conclusions in the paper are present in the paper and/or the Supplementary Materials. All methods, data and materials are available upon request. The raw mass spectrometry data has been uploaded onto the ProteomeXchange via the MassIVE (Mass Spectrometry Interactive Virtual Environment) partner repository with the dataset identifier PXD060401.

## References

[1] Lawrence, R., Watters, M., Davies, C. R., Pantel, K. & Lu, Y. J. Circulating tumour cells for early detection of clinically relevant cancer. *Nat. Rev. Clin. Oncol.* **20**, 487–500 (2023). 37268719 10.1038/s41571-023-00781-y PMC10237083

[2] Silberring, J. & Ciborowski, P. Biomarker discovery and clinical proteomics. *Trends Anal. Chem.* **29**, 128 (2010). 10.1016/j.trac.2009.11.007 PMC2822390 20174458

[3] Anderson, N. L. & Anderson, N. G. The human plasma proteome: history, character, and diagnostic prospects. *Mol. Cell Proteom.* **1**, 845–867 (2002). 10.1074/mcp.r200007-mcp200 12488461

[4] Tu, C. et al. Depletion of abundant plasma proteins and limitations of plasma proteomics. *J. Proteome Res.* **9**, 4982–4991 (2010). 20677825 10.1021/pr100646w PMC2948641

[5] Blume, J. E. et al. Rapid, deep and precise profiling of the plasma proteome with multi-nanoparticle protein corona. *Nat. Commun.* **11**, 3662 (2020). 32699280 10.1038/s41467-020-17033-7 PMC7376165

[6] Skog, J. et al. Glioblastoma microvesicles transport RNA and proteins that promote tumour growth and provide diagnostic biomarkers. *Nat. Cell Biol.* **10**, 1470–1476 (2008). 19011622 10.1038/ncb1800 PMC3423894

[7] Moon, P. G. et al. Fibronectin on circulating extracellular vesicles as a liquid biopsy to detect breast cancer. *Oncotarget* **7**, 40189–40199 (2016). 27250024 10.18632/oncotarget.9561 PMC5130002

[8] Niu, L. et al. Tumor-derived exosomal proteins as diagnostic biomarkers in non-small cell lung cancer. *Cancer Sci.* **110**, 433–442 (2019). 30407700 10.1111/cas.13862 PMC6317937

[9] Dixson, A. C., Dawson, T. R., Di Vizio, D. & Weaver, A. M. Context-specific regulation of extracellular vesicle biogenesis and cargo selection. *Nat. Rev. Mol. Cell Biol.* **24**, 454–476 (2023). 36765164 10.1038/s41580-023-00576-0 PMC10330318

[10] Huang, M. B. et al. Characterization of exosomes in plasma of patients with breast, ovarian, prostate, hepatic, gastric, colon, and pancreatic cancers. *J. Cancer Ther.* **10**, 382–399 (2019). 33833900 10.4236/jct.2019.105032 PMC8025783

[11] Sun, B. et al. Circulating exosomal CPNE3 as a diagnostic and prognostic biomarker for colorectal cancer. *J. Cell Physiol.* **234**, 1416–1425 (2019). 30078189 10.1002/jcp.26936

[12] Alegre, E. et al. Circulating melanoma exosomes as diagnostic and prognosis biomarkers. *Clin. Chim. Acta* **454**, 28–32 (2016). 26724367 10.1016/j.cca.2015.12.031

[13] Li, S. et al. Exosomal ephrinA2 derived from serum as a potential biomarker for prostate cancer. *J. Cancer* **9**, 2659–2665 (2018). 30087706 10.7150/jca.25201 PMC6072821

[14] Lucien, F. et al. MIBlood-EV: Minimal information to enhance the quality and reproducibility of blood extracellular vesicle research. *J. Extracell. vesicles* **12**, e12385 (2023). 38063210 10.1002/jev2.12385 PMC10704543

[15] Melvin, J. C. et al. Lipid profiles and risk of breast and ovarian cancer in the Swedish AMORIS study. *Cancer Epidemiol. Biomark. Prev.* **21**, 1381–1384 (2012). 10.1158/1055-9965.EPI-12-0188 22593241

[16] His, M. et al. Prospective associations between serum biomarkers of lipid metabolism and overall, breast and prostate cancer risk. *Eur. J. Epidemiol.* **29**, 119–132 (2014). 24519551 10.1007/s10654-014-9884-5

[17] Salmond, N., Khanna, K., Owen, G. R. & Williams, K. C. Nanoscale flow cytometry for immunophenotyping and quantitating extracellular vesicles in blood plasma. *Nanoscale* **13**, 2012–2025 (2021). 33449064 10.1039/d0nr05525e

[18] Didiasova, M. et al. STIM1/ORAI1-mediated Ca2+ influx regulates enolase-1 exteriorization. *J. Biol. Chem.* **290**, 11983–11999 (2015). 25805497 10.1074/jbc.M114.598425 PMC4424336

[19] Zang, H. Y., Gong, L. G., Li, S. Y. & Hao, J. G. Inhibition of alpha-enolase affects the biological activity of breast cancer cells by attenuating PI3K/Akt signaling pathway. *Eur. Rev. Med. Pharm. Sci.* **24**, 249–257 (2020). 10.26355/eurrev_202001_19917 31957838

[20] Zhang, J., Li, H., Miao, L. & Ding, J. Silencing of ENO1 inhibits the proliferation, migration and invasion of human breast cancer cells. *J. BUON* **25**, 696–701 (2020). 32521855

[21] Li, M., Li, J., Wang, J., Li, Y. & Yang, P. Serum level of anti-α-enolase antibody in untreated systemic lupus erythematosus patients correlates with 24-hour urine protein and D-dimer. *Lupus* **27**, 139–142 (2018). 28728510 10.1177/0961203317721752

[22] Graner, M. W. et al. Proteomic and immunologic analyses of brain tumor exosomes. *FASEB J.* **23**, 1541–1557 (2009). 19109410 10.1096/fj.08-122184 PMC2669426

[23] Didiasova, M., Schaefer, L. & Wygrecka, M. When place matters: shuttling of enolase-1 across cellular compartments. *Front. Cell Dev. Biol.* **7**, 61 (2019). 31106201 10.3389/fcell.2019.00061 PMC6498095

[24] Kang, H. J., Jung, S. K., Kim, S. J. & Chung, S. J. Structure of human alpha-enolase (hENO1), a multifunctional glycolytic enzyme. *Acta Crystallogr D. Biol. Crystallogr* **64**, 651–657 (2008). 18560153 10.1107/S0907444908008561

[25] Subramanian, A. & Miller, D. M. Structural analysis of alpha-enolase. Mapping the functional domains involved in down-regulation of the c-myc protooncogene. *J. Biol. Chem.* **275**, 5958–5965 (2000). 10681589 10.1074/jbc.275.8.5958

[26] Plow, E. F. & Das, R. Enolase-1 as a plasminogen receptor. *Blood* **113**, 5371–5372 (2009). 19478049 10.1182/blood-2009-03-208546

[27] Hsiao, K. C. et al. Surface alpha-enolase promotes extracellular matrix degradation and tumor metastasis and represents a new therapeutic target. *PLoS One* **8**, e69354 (2013). 23894455 10.1371/journal.pone.0069354 PMC3716638

[28] Wygrecka, M. et al. Enolase-1 promotes plasminogen-mediated recruitment of monocytes to the acutely inflamed lung. *Blood* **113**, 5588–5598 (2009). 19182206 10.1182/blood-2008-08-170837

[29] He, J. et al. Proteomic-based biosignatures in breast cancer classification and prediction of therapeutic response. *Int J. Proteom.* **2011**, 896476 (2011). 10.1155/2011/896476 PMC3202144 22110952

[30] Minic, Z. et al. Lysine acetylome of breast cancer-derived small extracellular vesicles reveals specific acetylation patterns for metabolic enzymes. *Biomedicines* **11**, 10.3390/biomedicines11041076 (2023). 10.3390/biomedicines11041076 PMC10135746 37189694

[31] Shi, Y. Y. et al. Association of enolase-1 with prognosis and immune infiltration in breast cancer by clinical stage. *J. Inflamm. Res* **16**, 493–503 (2023). 36785715 10.2147/JIR.S396321 PMC9922065

[32] Yuan, J. Q., Wang, S. M. & Guo, L. S100A9 promotes glycolytic activity in HER2-positive breast cancer to induce immunosuppression in the tumour microenvironment. *Heliyon* **9**, e13294 (2023). 36755606 10.1016/j.heliyon.2023.e13294 PMC9900376

[33] Delmonico, L. et al. Proteomic profile of saliva and plasma from women with impalpable breast lesions. *Oncol. Lett.* **12**, 2145–2152 (2016). 27602154 10.3892/ol.2016.4828 PMC4998569

[34] Yoon, K. H. et al. Comparative profiling by data-independent acquisition mass spectrometry reveals featured plasma proteins in breast cancer: a pilot study. *Ann. Surg. Treat. Res.* **106**, 195–202 (2024). 38586559 10.4174/astr.2024.106.4.195 PMC10995839

[35] Novais, A. A. et al. Exploring canine mammary cancer through liquid biopsy: proteomic profiling of small extracellular vesicles. *Cancers* **16**, 10.3390/cancers16142562 (2024). 10.3390/cancers16142562 PMC11275101 39061201

[36] Mohammed, S. I. et al. Tumor-draining lymph secretome en route to the regional lymph node in breast cancer metastasis. *Breast Cancer* **12**, 57–67 (2020). 32273752 10.2147/BCTT.S236168 PMC7104086

[37] Zarovni, N., Mladenovic, D., Brambilla, D., Panico, F. & Chiari, M. Stoichiometric constraints for detection of EV-borne biomarkers in blood. *J. Extracell. Vesicles* **14**, e70034 (2025). 39901737 10.1002/jev2.70034 PMC11791308

[38] Ferguson, S., Yang, K. S. & Weissleder, R. Single extracellular vesicle analysis for early cancer detection. *Trends Mol. Med.* **28**, 681–692 (2022). 35624008 10.1016/j.molmed.2022.05.003 PMC9339504

[39] Watts, S. E., Tunbridge, L. J., Smith, K. & Lloyd, J. V. Storage of platelets for tests of platelet function: effects of temperature on platelet aggregation, platelet morphology and liberation of beta-thromboglobulin. *Thromb. Res.* **44**, 365–376 (1986). 2948293 10.1016/0049-3848(86)90011-3

[40] Tassi Yunga, S. et al. Effects of ex vivo blood anticoagulation and preanalytical processing time on the proteome content of platelets. *J. Thromb. Haemost.* **20**, 1437–1450 (2022). 35253976 10.1111/jth.15694 PMC9887642

[41] Nieuwland, R. & Siljander, P. R. A beginner’s guide to study extracellular vesicles in human blood plasma and serum. *J. Extracell. Vesicles* **13**, e12400 (2024). 38193375 10.1002/jev2.12400 PMC10775135

[42] Tran, V. et al. Choice of blood collection methods influences extracellular vesicles counts and miRNA profiling. *J. Extracell. Biol.* **3**, e70008 (2024). 39440167 10.1002/jex2.70008 PMC11494683

[43] Xu, W. et al. Enolase 1 correlated with cancer progression and immune-infiltrating in multiple cancer types: a pan-cancer analysis. *Front. Oncol.* **10**, 593706 (2020). 33643901 10.3389/fonc.2020.593706 PMC7902799

[44] Huang, C. K., Sun, Y., Lv, L. & Ping, Y. ENO1 and cancer. *Mol. Ther. Oncolytics* **24**, 288–298 (2022). 35434271 10.1016/j.omto.2021.12.026 PMC8987341

[45] Almaguel, F. A., Sanchez, T. W., Ortiz-Hernandez, G. L. & Casiano, C. A. Alpha-enolase: emerging tumor-associated antigen, cancer biomarker, and oncotherapeutic target. *Front. Genet.* **11**, 614726 (2020). 33584813 10.3389/fgene.2020.614726 PMC7876367

[46] Scott, N. E. et al. Interactome disassembly during apoptosis occurs independent of caspase cleavage. *Mol. Syst. Biol.* **13**, 906 (2017). 28082348 10.15252/msb.20167067 PMC5293159

[47] Foster, L. J., De Hoog, C. L. & Mann, M. Unbiased quantitative proteomics of lipid rafts reveals high specificity for signaling factors. *Proc. Natl Acad. Sci. USA* **100**, 5813–5818 (2003). 12724530 10.1073/pnas.0631608100 PMC156283

[48] Rappsilber, J., Ishihama, Y. & Mann, M. Stop and go extraction tips for matrix-assisted laser desorption/ionization, nanoelectrospray, and LC/MS sample pretreatment in proteomics. *Anal. Chem.* **75**, 663–670 (2003). 12585499 10.1021/ac026117i

[49] Kerr, C. H. et al. Dynamic rewiring of the human interactome by interferon signaling. *Genome Biol.* **21**, 140 (2020). 32539747 10.1186/s13059-020-02050-y PMC7294662

[50] Beck, S. et al. The impact II, a very high-resolution quadrupole time-of-flight instrument (QTOF) for deep shotgun proteomics. *Mol. Cell. Proteom.* **14**, 2014–2029 (2015). 10.1074/mcp.M114.047407 PMC4587313 25991688

[51] Cox, J. & Mann, M. MaxQuant enables high peptide identification rates, individualized ppb-range mass accuracies and proteome-wide protein quantification. *Nat. Biotechnol.* **26**, 1367–1372 (2008). 19029910 10.1038/nbt.1511

[52] Cox, J. et al. Accurate proteome-wide label-free quantification by delayed normalization and maximal peptide ratio extraction, termed MaxLFQ. *Mol. Cell. Proteom.* **13**, 2513–2526 (2014). 10.1074/mcp.M113.031591 PMC4159666 24942700

[53] Choi, M. et al. MassIVE.quant: a community resource of quantitative mass spectrometry-based proteomics datasets. *Nat. Methods* **17**, 981–984 (2020). 32929271 10.1038/s41592-020-0955-0 PMC7541731

[54] Schwanhausser, B. et al. Corrigendum: Global quantification of mammalian gene expression control. *Nature* **495**, 126–127 (2013). 23407496 10.1038/nature11848

