# Supplementary material for: Circulating enolase 1 as a diagnostic biomarker for early-stage breast cancer
Source: NPJ Precis Oncol. 2025 Oct 17;9:326. doi: 10.1038/s41698-025-01109-y (PMC12534435; doi:10.1038/s41698-025-01109-y)
Supplement: Supplementary file 1 — Supplementary Information [file 41698_2025_1109_MOESM1_ESM.pdf]

Supplementary Information for

Circulating Enolase 1 as a Diagnostic Biomarker for Early-Stage Breast Cancer

Nikki Salmond et al.
*Corresponding author: Dr. Karla Williams. Email karla.williams@ubc.ca

This PDF file includes:
Figs. S1 – S7
Tables S1 – S4

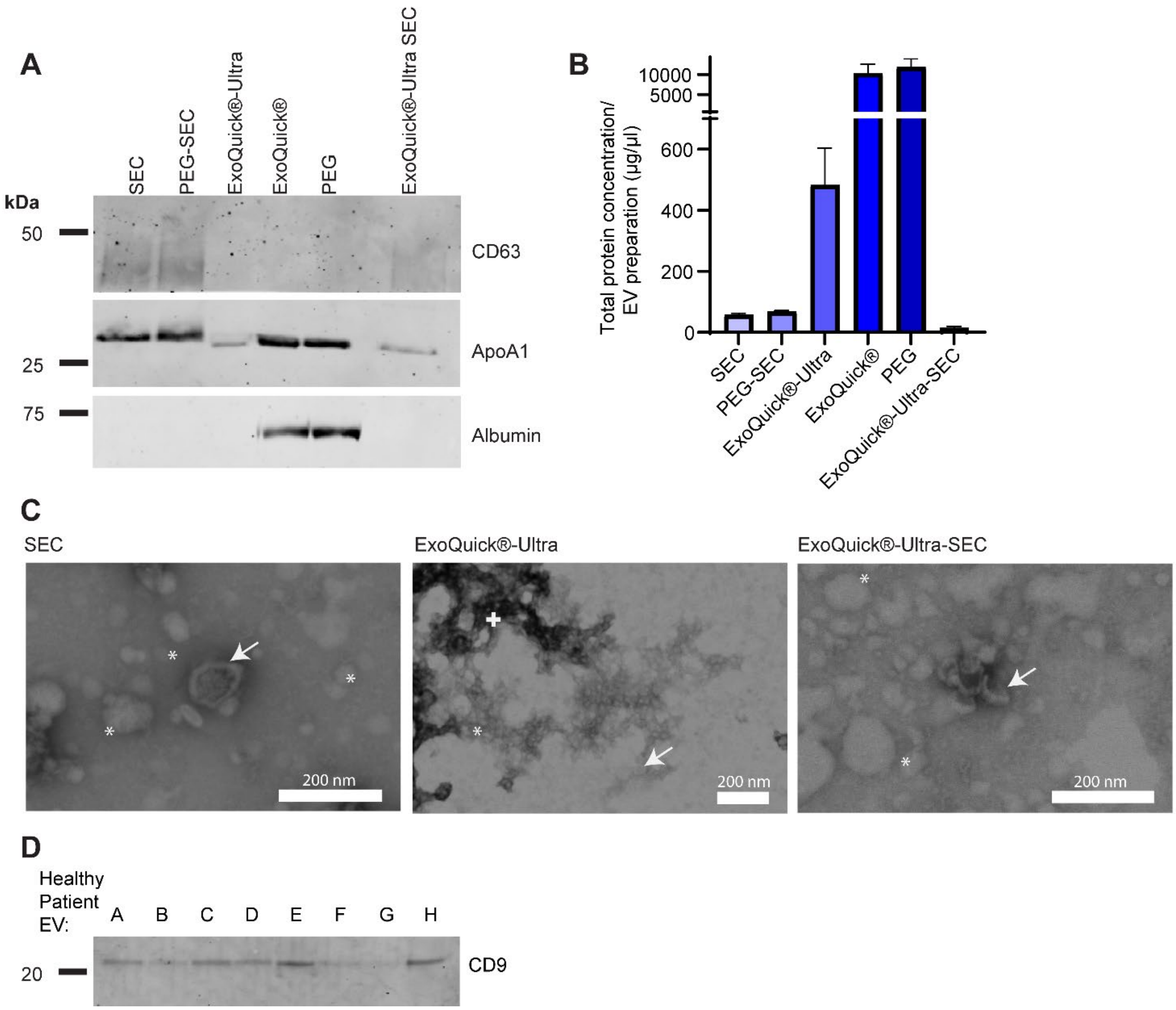

Supplementary Figure 1: EV isolation optimization using size exclusion chromatography, precipitation, and ExoQuick®-Ultra or a combination thereof. (A) EVs were isolated from 500 µl plasma by SEC, precipitation, ExoQuick®-Ultra, or a combination of techniques. Resultant EVs (20 µg) were Western blotted for EV marker CD63, lipoprotein contaminant ApoA1 and plasma contaminant albumin. Un-cropped blots can be found in Supplementary Figure 2. (B) The protein concentration of each EV preparation was quantified using a micro-BCA assay. (C) Scanning Transmission electron microscopy was used to visualize EVs isolated by SEC, ExoQuick®-Ultra and ExoQuick®-Ultra-SEC. Arrows = EV, * = lipoproteins and + = protein. D) Western blot of 10 µg of eight random healthy patient plasma-derived SEC isolated EV samples for a second EV marker - CD9.

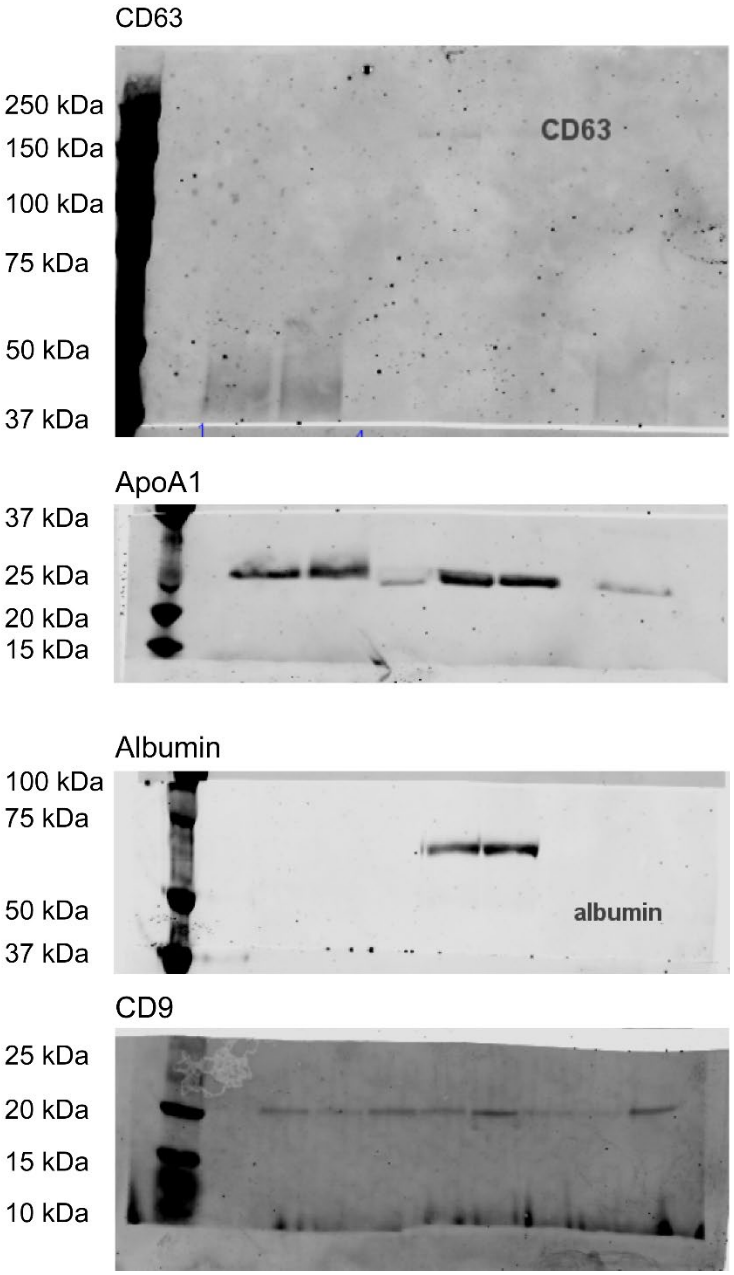

Supplementary Figure 2: Up-cropped Western blots from Supplementary figure 1.

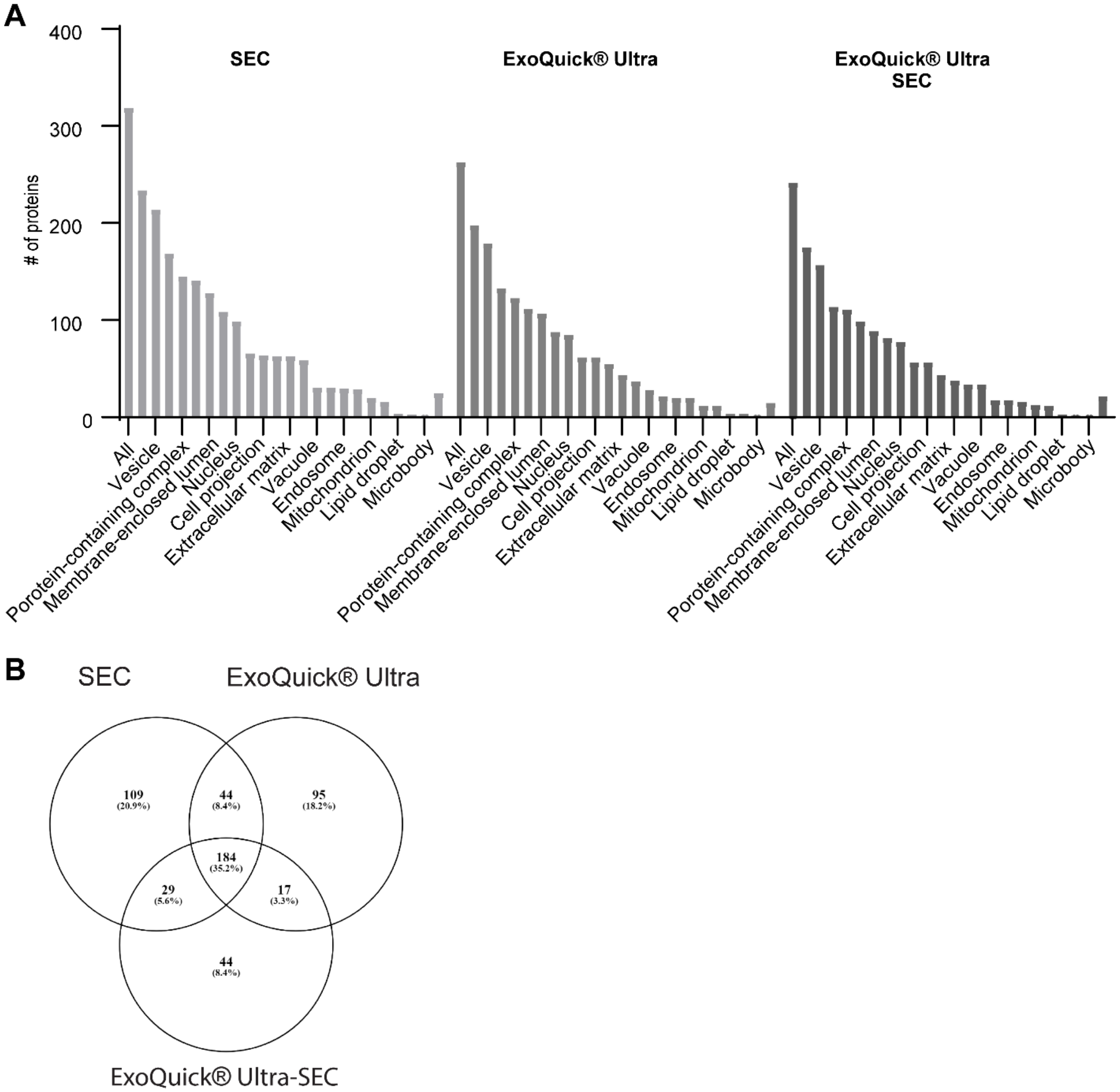

Supplementary Figure 3: Mass spectrometry analysis of EV proteomes after isolation by SEC, ExoQuick®-Ultra and ExoQuick®-Ultra-SEC. (A) Proteins found in EV preparations identified by mass spectrometry were subjected to Go-Analysis (WebGeSalt, 2024). The cellular compartment from which the proteins were derived was analyzed. (B) Venny (2024) was used to construct a Ven Diagram to represent the unique and common identified proteins in EV preparations isolated by each technique.

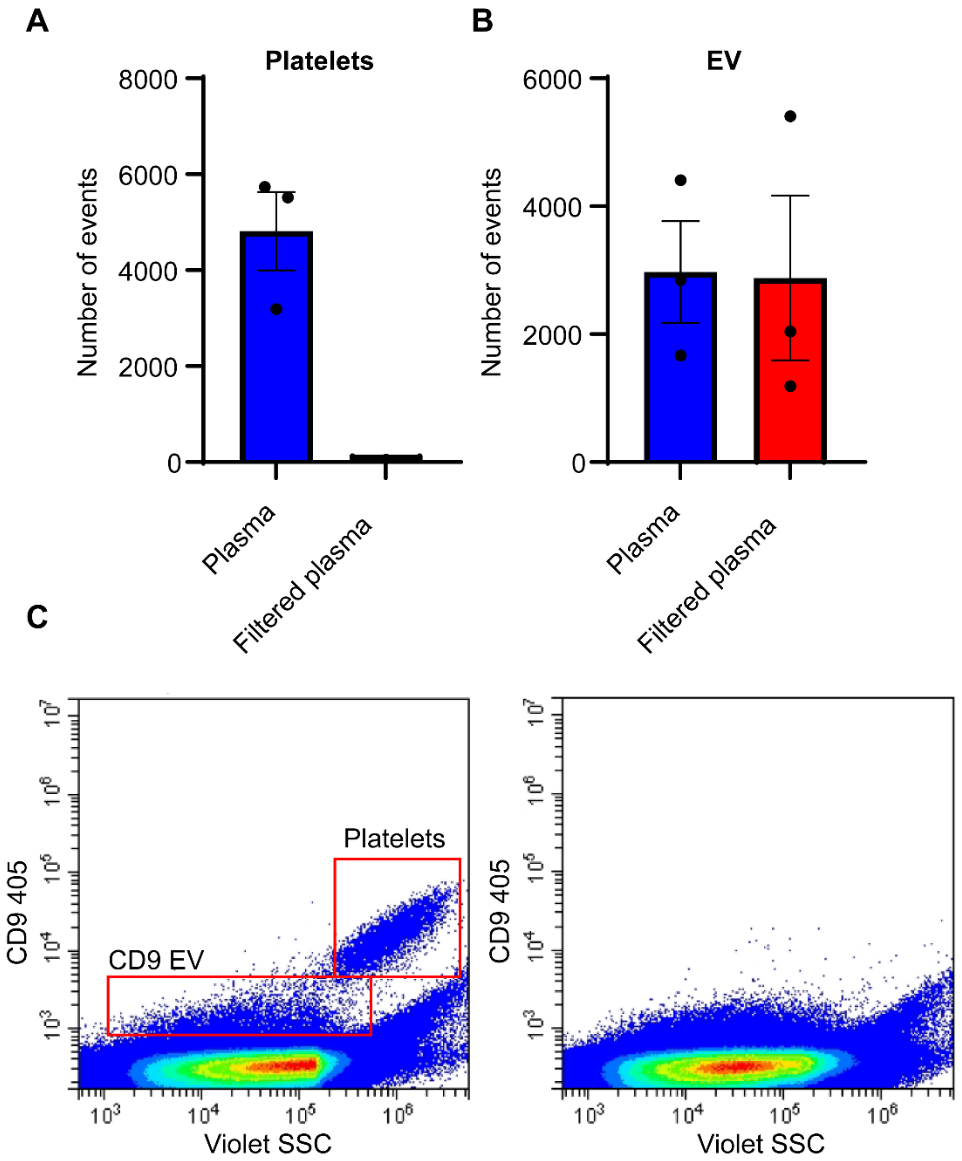

Supplementary Figure 4: Filtration of plasma removes platelets while retaining EVs. (A) Nanoscale flow cytometry shows CD9 positive platelets are removed by 0.8 μm plasma filtration while (B) retaining CD9 positive EVs. C) Representative images of platelet and EV populations. N=3 individual patient plasma samples. +/- SEM.

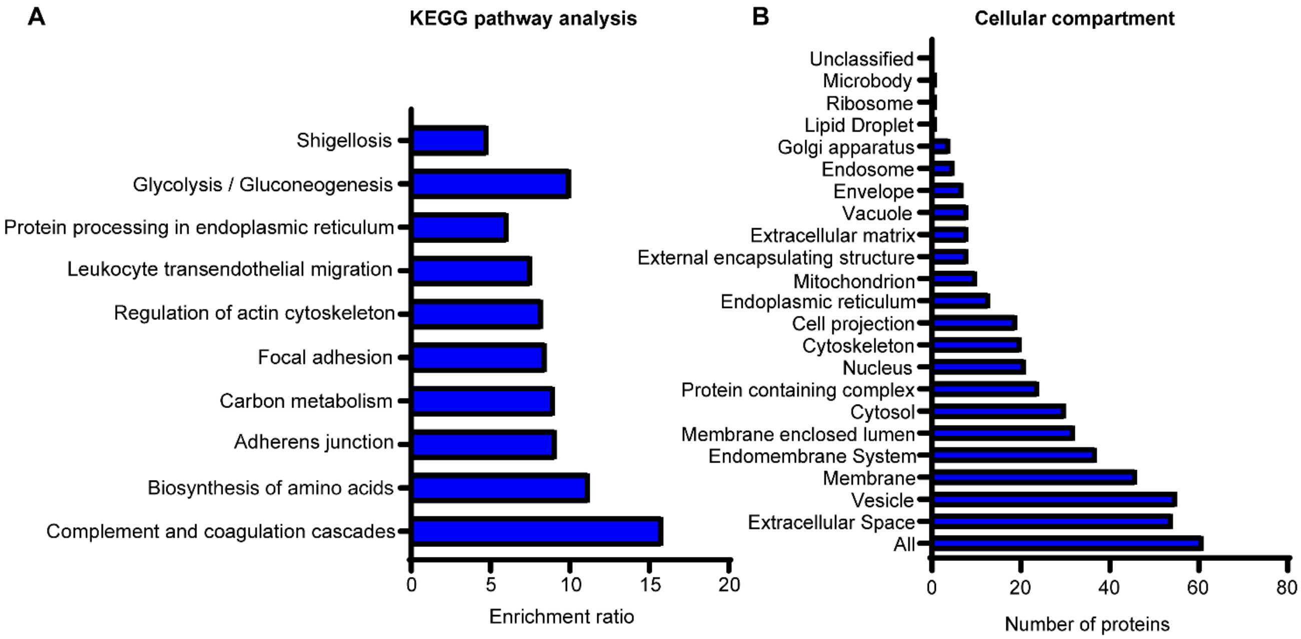

Supplementary Figure 5: KEGG pathway analysis of significantly elevated proteins in breast cancer. WebGeStalt (2024) was used to carry out KEGG pathway analysis of the biological pathways that significantly elevated proteins in breast cancer patient plasma are often involved in, and Go-analysis was used to identify the cellular compartments in which the proteins are associated with.

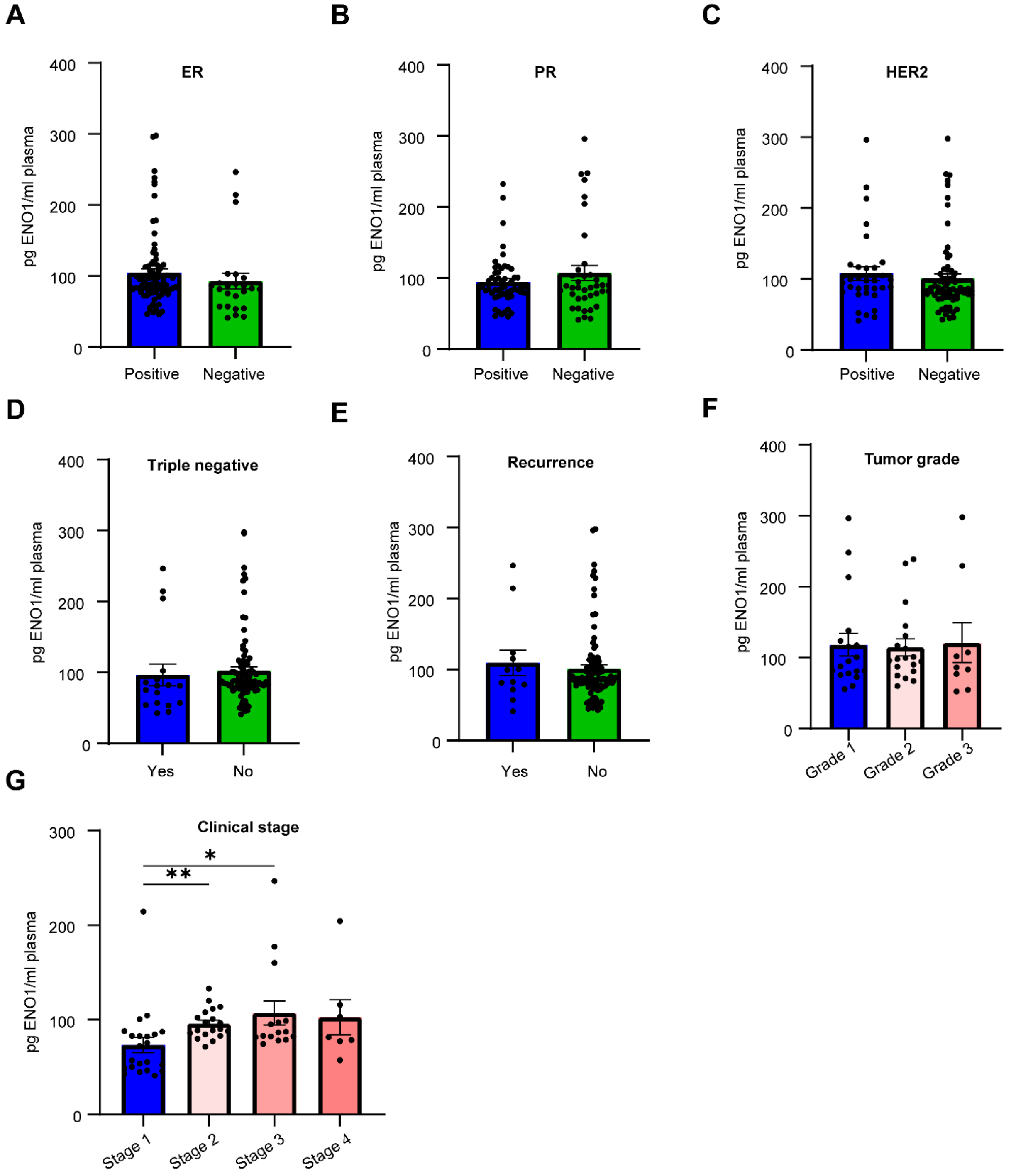

Supplementary figure 6: Enolase 1 levels in breast cancer patient samples is not predictive of hormone status, tumor grade, clinical grade, or recurrence. ELISA was used to quantify Enolase 1 in breast cancer patient plasma samples and the data was further analyzed taking into consideration the specifics of each individual patients diagnosis: estrogen receptor positivity (A), progesterone receptor positivity (B), HER2 positivity (C), triple negative (D), recurrence (E), tumor grade (F), and clinical stage (G).

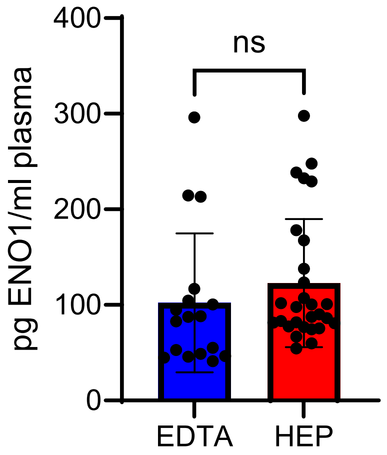

Supplementary figure 7: The anticoagulant used in plasma preparation does not affect enolase 1 ELISA results: Graph shows the amount of plasma enolase 1 (pg/ml) detected in breast cancer (BC Cancer and London biobanks excluding pre-operative) patient samples by ELISA. EDTA N = 17. Heparin N = 28.

Supplementary Table 1: The top 100 EV-associated proteins as identified by multiple published studies (Vesiclepedia, 2024) also detected in EV preparations isolated by SEC, ExoQuick®-Ultra and ExoQuick®-Ultra-SEC after analysis by mass spectrometry.

EV protein	SEC	ExoQuick®-ULTRA	ExoQuick®-Ultra-SEC
CD63	-	-	-
CD9	y	-	-
PDCD6IP	y	-	-
TSG101	-	-	-
CD81	-	-	-
GAPDH	y	y	y
FLOT1	y	-	-
ACTB	y	y	y
ANXA2	-	y	-
SDCBP	y	-	-
HSP90AA1	-	-	-
HSPA8	y	y	-
ANXA5	-	-	y
ENO1	y	y	y
PKM	y	y	y
HSP90AB1	y	-	y
YWHAZ	y	y	y
PGK1	-	-	-
FLOT2	Y	-	-
YWHAE	y	y	y
ANXA1	-	-	y
VCP	y	-	-
PPIA	y	-	-
EEF1A1	-	-	y
ALB	y	y	y
ALDOA	y	-	y
ITGB1	y	-	-
MYH9	y	-	y
CLTC	-	-	-
TPI1	y	-	-
CFL1	y	y	y
EEF2	-	-	-
MSN	y	-	-
GNB1	-	-	-
PRDX1	y	-	-
SLC3A2	-	y	-
ANXA6	-	-	-
EZR	-	-	-
LDHA	-	y	-
LDHB	-	y	-
CDC42	y	-	-
BSG	-	-	-
PFN1	y	y	y
ATP1A1	-	-	-
ACTN4	-	-	-
HSPA1A	-	-	-
YWHAB	-	-	y
FLNA	Y	Y	Y
GNAI2	y	-	-
YWHAQ	-	-	-
FASN	-	-	-
CLIC1	-	-	-
PRDX2	Y	-	-
GSN	Y	Y	Y
RAP1B	Y	Y	-
CCT2	-	-	-
RAB5C	-	-	-
HIST1H4A	-	-	-
LGALS3BP	Y	Y	Y
GNB2	-	-	-
YWHAG	Y	-	-
RAB10	Y	-	-
HLA-A	-	-	-
ANXA7	-	-	-
ACTN1	-	Y	Y
FN1	Y	Y	Y
TFRC	Y	Y	Y
RAN	-	-	-
GDI2	-	-	-
CCT3	-	-	-
AHCY	-	-	-
HSPA5	Y	Y	Y
CCT4	-	-	-
ACLY	-	-	-
C3	Y	Y	Y
UBA1	-	-	-
ANXA11	-	-	-
KPNB1	-	-	-
CAP1	Y	-	-
TUBB4B	Y	-	-
RAC1	-	-	-
MFGE8	-	-	-
TCP1	-	-	-
RHOA	-	-	-
TLN1	Y	Y	Y
GNAS	-	-	-
CCT6A	-	-	-
CCT5	-	-	-
RALA	-	-	-
EHD1	-	-	-
CCT8	-	-	-
PGAM1	-	-	-
VCL	Y	Y	-
IQGAP1	-	-	-
EIF4A1	-	-	-
GPI	-	-	-
RAB7A	-	-	-
EEF1G	-	-	-
ADAM10	-	-	-
A2M	Y	Y	Y
**Total top 100 markers**	**42**	**26**	**26**
**Other EV proteins of interest**			
PDCD6IP (ALIX)	Y	-	-
SDCBP (Syntenin)	Y	-	-
Rab11B	Y	-	-
Rab11A	Y	-	-
Rab10	Y	-	-
Rab27B	Y	-	-
ITGB3	Y	-	-
ITGA2B	Y	-	-
ITGB1	Y	-	-
ITGA2	Y	-	-
FLOT1	Y	-	-
**Total**	**53**	**26**	**26**

Supplementary table 2: Mass spectrometry clinical cohort information

Cohort	Number	Gender	Age range	Pre/peri/post menopause
**Healthy**	19	Female	48-69	3/2/14
**Benign**	19	Female	36-82	8/0/7*
**Stage 1 Breast cancer**	77	Female	27-88	10/2/64**
**Pre & post op**	9	Female	46-82	1/1/7

*4 unknown menopausal status in benign cohort

** 1 unknown menopausal status in breast cancer cohort

Supplementary Table 3: Mass spectrometry identified potential early-stage breast cancer biomarkers and their associated p-values

Protein	P value
**Higher in Breast cancer**	
MYH9	<0.000001
KNG1	0.000001
C1S	0.000001
F11	<0.000001
SERPINC1	0.000021
MYL12A;MYL12B	0.000003
HRG	0.000041
ITGA2B	0.000014
F5	0.000063
GNB1;GNB2	0.000069
HSPA5	0.000103
ITGB3	0.000178
SOD2	0.000051
CFL1;CFL2	0.000107
KLKB1	0.000249
TMSB4X	0.00033
VCL	0.001269
ACTC1;ACTG2;ACTA2;ACTA1	0.001595
TLN1	0.002007
FERMT3	0.001179
PFN1	0.001964
FLNA	0.002934
TPM4	0.001293
ILK	0.001716
CAP1	0.000954
MYL9	0.000515
GNAI2;GNAI1	0.00668
UBA52;RPS27A;UBB;UBC	0.00684
RAB1B;RAB1C	0.003228
PDIA3	0.000811
ITGA6	0.002735
SUCLG1	0.011375
SDPR	0.003699
ACTB;ACTG1	0.017403
YWHAZ	0.012549
TTR	0.021099
MMRN1	0.02034
HSPA8	0.017542
C1R	0.024222
GAPDH	0.025403
RAP1B;RAP1A	0.030222
STOM	0.012628
MSN	0.031635
YWHAE	0.005292
PPIB	0.00373
CAMP	0.040642
VTN	0.015448
ENO1	0.029717
PTPRJ	0.004273
ACTN1	0.004283
PECAM1	0.008072
SH3BGRL3	0.011238
PPBP	0.006274
ATP2A3	0.004308
HSP90B1	0.020544
SRC	0.017724
ATP5B	0.013864
ATP5A1	0.005322
PKM	0.025816
RSU1	0.011088
CALR	0.020102
VDAC3	0.008221
CD9	0.027783
TAGLN2	0.040878
CLTC	0.036816
PGK1	0.021981
CD47	0.035458
RALB	0.03637
WDR1	0.042483
P4HB	0.017623
RAB27B	0.046897
HSPE1	0.034134
IDH2	0.02639
**Lower in breast cancer**	
SUZ12	0.004773
GPLD1	0.017919
ANPEP	0.035857
IGKV1-5	0.048513
SELL	0.033438
PON1	0.049269
APOA4	0.048686
IGLV3-10;IGLV3-16	0.039954
C4BPA	0.037805
FGB	0.037372
GSR	0.031071
AHSG	0.019
ITIH1	0.018388
CFH	0.01336
C4B	0.011827
HBD	0.006351
FGA	0.00556
FCN3	0.000562
SERPING1	0.000457

Supplementary Table 4: Pre-operative and post-operative (after tumor removal) blood draw information.

Pre-operative & post-operative sample	Days after surgery: second blood draw *Mass Spec*	Days after surgery: second blood draw *ELISA*
**1**	66	54
**2**	62	36
**3**	27	64
**4**	41	26
**5**	51	60
**6**	44	52
**7**	40	43
**8**	25	51
**9**	41	52
**10**		56
**11**		38
**12**		27
**13**		48
**14**		39
